# Extracorporeal shockwave therapy for degenerative meniscal tears results in a decreased T2 relaxation time and pain relief: An exploratory randomized clinical trial

**DOI:** 10.1002/ksa.12384

**Published:** 2024-08-05

**Authors:** Shogo Hashimoto, Takashi Ohsawa, Hiroaki Omae, Atsufumi Oshima, Ryota Takase, Hirotaka Chikuda

**Affiliations:** ^1^ Department of Orthopaedic Surgery Gunma University Graduate School of Medicine Maebashi Gunma Japan; ^2^ Department of Orthopaedic Surgery Zenshukai Hospital Maebashi Gunma Japan; ^3^ Department of Orthopaedic Surgery Takasaki Genaral Medical Center Takasaki Gunma Japan

**Keywords:** extracorporeal shockwave therapy, meniscus healing, meniscus tear, MRI, T2 mapping

## Abstract

**Purpose:**

The optimal management of degenerative meniscal tears remains controversial. Extracorporeal shockwave therapy (ESWT) has been shown to promote tissue repair in both preclinical and clinical studies; however, its effect on degenerative meniscal tears remains unknown. This study aimed to examine whether ESWT improves meniscal degeneration.

**Methods:**

This randomized trial was conducted between 2020 and 2022 and involved patients with degenerative medial meniscal tears. Patients were allocated to receive either focused ESWT (0.25 mJ/mm^2^, 2000 impulses, 3 sessions with a 1‐week interval) or sham treatment. Patients were evaluated using magnetic resonance imaging (MRI) before treatment and at 12 months after treatment. The primary endpoint was improvement in meniscal degeneration, as assessed by the change in T2 relaxation time from baseline on MRI T2 mapping. Knee pain and clinical outcomes were also examined at the same time.

**Results:**

Of 29 randomized patients, 27 patients (mean age 63.9 ± 8.7 years; females 37%; ESWT group 14 patients; control group 13 patients) were included in the final analysis. At 12 months postintervention, patients in the ESWT group showed a greater decrease in the T2 relaxation time (ESWT group −2.9 ± 1.7 ms vs. control group 1.0 ± 1.9 ms; *p* < 0.001) and had less knee pain (*p* = 0.04). The clinical outcomes at 12 months post‐treatment were not statistically significant. No adverse events were reported.

**Conclusion:**

ESWT decreased the T2 relaxation time in the meniscus at 12 months post‐treatment. ESWT also provided pain relief, but no differences were observed in clinical outcomes.

**Level of Evidence:**

Level II.

**Abbreviations**
ADL: activities of daily living
CCN2: cellular communication network factor 2
CI: confidence interval
ESWT: extracorporeal shockwave therapy
IKDC: International Knee Documentation Committee
K–L: Kellgren–Lawrence
KOOS: knee injury and osteoarthritis outcome score
LIPUS: low‐intensity pulsed ultrasound
MRI: magnetic resonance imaging
MSCs: mesenchymal stem cells
NRS: numerical rating scale
PROs: patient‐reported outcomes
PS: performance status
SD: standard deviation
SOX9: SRY‐box transcription factor 9

## INTRODUCTION

Extracorporeal shockwave therapy (ESWT) involves the delivery of an acoustic shock wave to a specific area of the body. ESWT has been used to treat various orthopaedic disorders, including tendonitis, enthesopathy, calcification and pseudoarthrosis [12, 42]. Previous studies have reported that low‐energy ESWT is minimally invasive, repeatable and effective by promoting biological processes such as tissue regeneration, bone remodelling, anti‐inflammation and cartilage protection [6, 10, 17, 37]. In bone and cartilage, ESWT stimulates both progenitor and differentiated cells, and mechanical pressure on the cells has a positive effect on the pathology by increasing the expression of cell‐specific proteins and cell viability [46]. Furthermore, the clinical utility of ESWT in knee osteoarthritis has been demonstrated [22, 40]. Regarding the effect of ESWT on meniscal tears, it was previously demonstrated that the tissue repair of medial meniscal tears was promoted by ESWT, and the degenerated medial meniscus was observed to be protected in a rat model [13, 39]. Despite its potential therapeutic effect on the human meniscus, clinical studies examining the effects of ESWT on degenerative meniscal tears are lacking.

Degeneration of the meniscus is characterized by a disorganized collagen network, decreased proteoglycan levels and increased water content [26, 44]. While conventional magnetic resonance imaging (MRI) has high sensitivity and specificity to detect meniscal tears [5, 35], it has limited utility in assessing degeneration of the meniscus [28, 45]. Recently, quantitative MRI has been increasingly utilized to obtain information on the composition of specific tissues of interest [3, 27, 31]. In particular, T2 mapping enables the evaluation of the meniscal condition by measuring the T2 relaxation time, which can detect water content, composition and anisotropic changes in collagen fibres [1, 9, 23, 24]. T2 mapping has been shown to have a correlation with the degree of histological degeneration [9, 29] and has been utilized in clinical studies [3, 30, 47]. Therefore, the T2 relaxation time was focused on in this study, as it is considered a compositional biomarker for quantifying meniscus degeneration.

There is still no established treatment for degenerative meniscus, and this remains a clinical challenge [25]. In particular, since few treatments have been found to achieve any biological improvements, the tissue repair effects of ESWT were focused on in this study. The purpose of this exploratory randomized trial was to examine whether ESWT has a biological therapeutic effect on degenerative meniscal tears by evaluating the T2 relaxation time. It was hypothesized that a decrease in the T2 relaxation time in the degenerated meniscus would be observed at 12 months after the intervention with ESWT.

## MATERIALS AND METHODS

### Trial design

The trial protocol was approved by the Gunma University ethics committee　(IRB2020020‐001) and was registered in the Japan Registry of Clinical Trials (jRCTs032200082). This was a placebo‐controlled (specifically, sham‐controlled), single‐centre, randomized clinical trial with a 1:1 parallel group. This study, which included symptomatic patients with degenerative meniscal tears, was conducted between 2020 and 2022. All participants were informed of the purpose and content of the study, and they provided written informed consent. We followed the Consolidated Standards of Reporting Trials (CONSORT) guidelines.

### Participants

Patients who were 40 years of age or older with degenerative meniscal tears were included in this study (Table 1). Any patients with advanced knee osteoarthritis (Kellgren–Lawrence grade 3 or 4) were excluded. Prior to enrollment, patients were assessed for physical findings and underwent routine knee radiography and conventional MRI to evaluate meniscal tears. The principal investigator (S. H.) assessed patient eligibility. After enrollment, the patients were randomly assigned to receive ESWT or sham treatment using a software programme (HOPE eACReSS).

**Table 1:** Inclusion and exclusion criteria.

Inclusion criteria
Age ≥40 years
Degenerative meniscus tear of the medial posterior segment diagnosed by MRI
Knee pain with a NRS of ≥2
PS 0–1
Treatment on an outpatient basis
Exclusion criteria
Osteoarthritis classified as ≥K‐L G3
Cognitive impairment
Untreated haemophilia or other blood coagulation disorder or taking anticoagulants
History of thrombosis
Complications of malignancy
Pregnant
>6 months of steroid therapy or within 6 weeks after steroid injection

Abbreviations: K–L, Kellgren–Lawrence; MRI, magnetic resonance imaging; NRS, numerical rating scale; PS, performance status.

### Intervention

In this study, an ESWT device (DUOLITH® SD1; STORZ MEDICAL) was used. Intervention protocols were determined based on a previous study [12]. The total energy flux density was increased continuously from 0.01 to 0.25 mJ/mm^2^ within 500 introductory impulses. Thereafter, 2000 treatment impulses with 0.25 mJ/mm^2^ (four impulses per second) were administered per session, and the intervention was repeated up to a total of three sessions in weekly intervals. The intervention group felt some pain with an increase in energy, but no patient was unable to tolerate the pain, and all were able to increase their energy to 0.25 mJ/mm^2^.

In the control group, a placebo pad was used to prevent the shock waves from reaching the patient. The control group was given the same sham intervention with the same standoff as in the previous study [12], prepared using STORZ MEDICAL. This standoff was filled with air, and the shock wave could not transmit air; thus, no energy could reach the patient. The placebo handpieces were identical in design, shape and weight; therefore, participants were unable to identify the placebo handpieces. Patients were not informed of their respective procedures during the study period.

Before shockwave delivery, the location of the posterior segment of the meniscus was identified using ultrasonography in the prone position to determine the precise location of the target. Since treating only the precise site of the meniscus is difficult, a slightly wider area around the injured meniscus was treated. Both ESWT and the sham treatment were performed in the same manner by the authors (S. H. and H. O.).

In this study, we did not standardize the rehabilitation therapy and medication because there was no established clinical bundle. Patients in both groups received regular outpatient instructions regarding their daily activities or sports participation.

### Primary outcome (T2 mapping)

The primary endpoint was the change in T2 relaxation time on MRI T2 mapping from baseline to 12 months after treatment. T2 mapping enables the assessment of the degree of meniscal degeneration with high sensitivity and specificity. The more severe the meniscal degeneration, the longer the T2 relaxation time [29].

**Figure 1:**
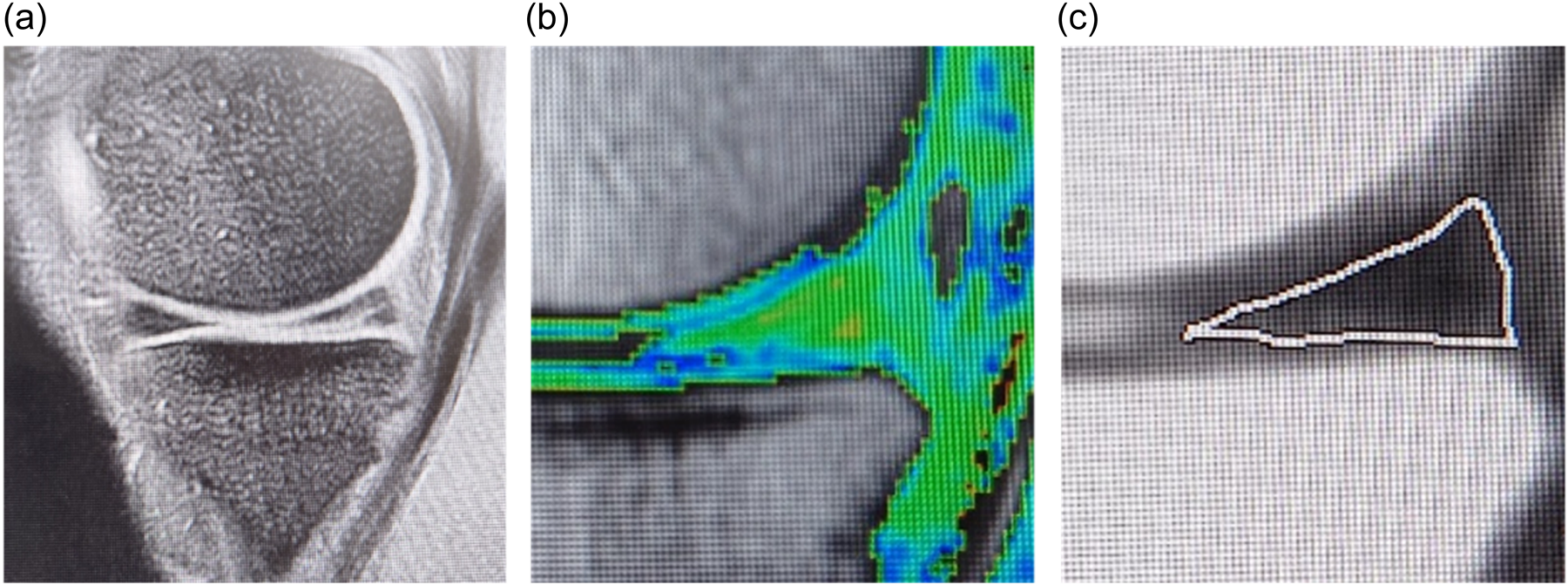
Region of interest for T2 mapping of meniscus tears. (a) Overview of the medial meniscus in the sagittal plane on the T2* sequence. (b, c) Magnification of the white square shown in (a). (b) T2 colour mapping. The warm‐coloured region on T2 colour mapping that is coincident with the region of signal change at the meniscal injury on conventional magnetic resonance imaging was defined as the region of interest for this patient. (c) The area surrounded by the line indicates the whole meniscus. T2 relaxation times were measured on T2 mapping.

For T2 mapping, a Signa HDxt 1.5 T MRI machine (GE Healthcare) and a Transmit/Receive 8 Channel Knee Coil with a T2 mapping sequence were used (repetition time = 1200 ms; echo time = 8 echoes (8–63.6 ms); flip angle = 90°; bandwidth = 244 Hz/pixel; field of view = 150 × 150 mm; matrix size = 256 × 160 pixels; voxel size = 0.6 × 0.9 × 4.0 mm). The region of interest for the measurement of T2 relaxation times was set according to the area of the injured meniscus showing high signal intensity on The T2* sequence acquired at the same time (Figure 1) [47]. The T2 relaxation time was measured as the average of the entire meniscus at the site of the tear. Each patient was measured at the same site at eligibility assessment and at 6 and 12 months after the intervention. To evaluate the measurement accuracy of the T2 relaxation time, the standard error of measurement (SEM) was calculated by performing five measurements on the same subject. To calculate the inter‐ or intra‐class coefficient (ICC), the T2 relaxation time was measured by knee surgeons with >10 years of experience, who were blinded to the patient allocation, by reading 30 selected films twice in 2 weeks.

### Other outcomes

Patients were also evaluated for the following outcomes: numerical rating scale (NRS) for knee pain, the use of analgesics such as non‐steroidal anti‐inflammatory drugs (NSAIDs) or acetaminophen at least once a day for meniscus injuries, physical findings (range of motion [ROM], presence of patellar floating, McMurray test) and patient‐reported outcomes (PROs), including the International Knee Documentation Committee [IKDC] subjective knee evaluation form, Lysholm score and knee injury and osteoarthritis outcome score [KOOS] at each outpatient visit (each treatment session, 3, 6 and 12 months after treatment).

### Adverse events

Adverse events were defined as unexpected events attributable to the allocated treatment. We assessed whether patients experienced any adverse events from their intervention at each outpatient visit during the study period.

### Statistical analysis

For continuous variables, the normality of the distribution was tested using Levene's test. Independent *t*‐tests and chi‐square tests were performed to assess between‐group differences in the cross‐sectional evaluation of the secondary outcomes. For longitudinal analysis of the primary outcome, a paired‐sample *t*‐test was performed. Data are expressed as mean ± SD. For all statistical analyses, significance was set at *p* < 0.05. All statistical analyses were performed using the IBM SPSS Statistics version 26 for Windows.

The sample size was calculated using a software programme (G*Power 3.1, HHU). Based on a previous study [24], it was assumed that the mean T2 relaxation time in a meniscus with degenerative tears was approximately 3.0 ms higher than that in a normal meniscus, with a standard deviation of 3.0 ms (estimated effect size, 1.0). A power analysis indicated that a total sample size of 16 patients (eight patients in each cohort) would provide 80% power (*β* = 0.20, *α* = 0.05) to detect a difference in T2 relaxation time. Accordingly, we planned to include 15 knees per group to account for any potential dropout.

## RESULTS

### Patient characteristics

**Figure 2:**
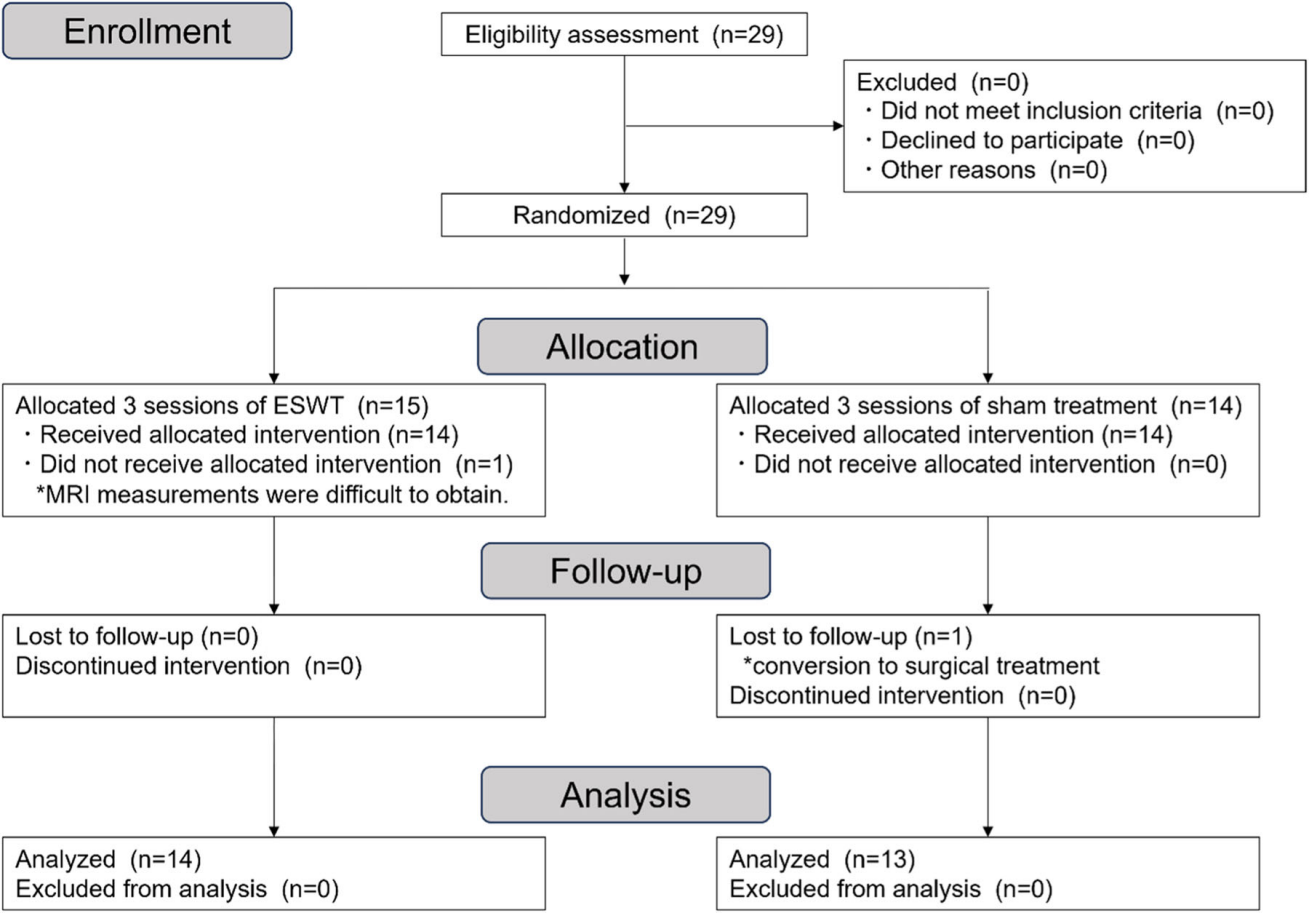
Consolidated Standards of Reporting Trials (CONSORT) diagram. Flow diagram of enrollment, allocation, follow‐up and analysis showing patient exclusion over the course of the study. ESWT, extracorporeal shockwave therapy.

A total of 29 patients underwent randomization between 2020 and 2021 (Figure 2). One patient assigned to the ESWT group was excluded immediately after randomization because of a meniscus injury, which precluded further evaluation of MRI findings. Another patient in the control group underwent surgery after a 6‐month evaluation and was excluded. Consequently, 27 patients were included in the final analysis (mean age, 63.9 ± 8.7 years; women 11 and men 16). All patients, except those excluded during the study period, completed follow‐up. The baseline characteristics of the two groups were similar (Table 2), with no differences in sex, age, height, weight, body mass index, comorbidities, sports activities, smoking history, drinking history, duration of symptoms, Mink grade 3, or K–L classification. The common comorbidities were hypertension in nine patients, dyslipidemia in five, hyperuricemia in four, mild diabetes mellitus in two and cardiac disease in two.

**Table 2:** Patient characteristics.

	ESWT group (*n* = 14)	Control group (*n* = 13)
Sex		
Female	5 (36%)	6 (46%)
Male	9 (64%)	7 (54%)
Age^a^ (years)	65.5 ± 9.0	63.5 ± 7.0
Height^a^ (cm)	163.0 ± 9.4	162.4 ± 9.7
Weight^a^ (kg)	63.6 ± 12.0	63.6 ± 9.3
BMI^a^ (kg/m^2^)	23.8 ± 3.3	24.2 ± 2.6
Comorbidities	2 (14%)	4 (31%)
Sports activities	6 (42%)	4 (31%)
Duration from onset^a^ (months)	6.2 ± 4.9	6.7 ± 4.5
Mink grade (0/1/2/3A/3B)	0/0/2/6/6	0/0/2/4/7
K–L classification (0/1/2/3/4)	5/7/2/0/0	3/6/4/0/0
Femorotibial angle	176.6 ± 1.9	176.6 ± 3.7
ROM extension^a^ (°)	−0.7 ± 1.8	−2.1 ± 3.2
ROM flexion^a^ (°)	136.8 ± 9.1	135.4 ± 4.5
Patellar floating	1 (7%)	3 (23%)
McMurray test	9 (64%)	12 (92%)
IKDC^a^	59.9 ± 17.9	53.8 ± 10.4
Lysholm score^a^	82.1 ± 10.8	65.3 ± 14.0
KOOS		
Symptoms^a^	74.5 ± 20.5	72.2 ± 16.1
Paina	76.0 ± 15.3	63.5 ± 7.0
ADL^a^	85.5 ± 13.5	77.7 ± 9.9
Sports/Rec^a^	58.2 ± 23.3	47.3 ± 21.1
QOL^a^	48.7 ± 14.1	43.7 ± 14.1

Abbreviations: ADL, activities of daily living; BMI, body mass index; ESWT, extracorporeal shockwave therapy; IKDC, International Knee Documentation Committee; K–L, Kellgren–Lawrence; KOOS, knee injury and osteoarthritis outcome score; QOL, quality of life; ROM, range of motion.

^a^ Values are presented as mean ± SD.

### Primary outcome: MRI T2 mapping

**Figure 3:**
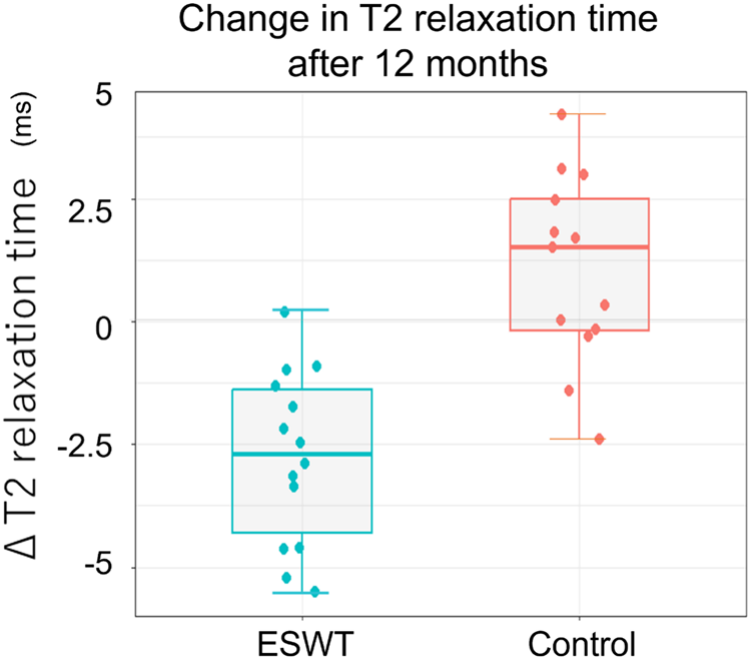
Change in T2 relaxation time after 12 months. Graph showing the box and whisker plot in T2 relaxation time for magnetic resonance imaging T2 mapping 12 months after intervention. The ESWT group had a significantly greater reduction in T2 relaxation time than the control group at 12 months (ESWT group −2.9 ± 1.7 ms vs. control group 1.0 ± 1.9 ms; *p* < 0.001). ESWT, extracorporeal shockwave therapy.

All 27 participants underwent T2 mapping 6 and 12 months post‐intervention. The T2 relaxation time was measured, and the SEM was 0.31 ms, and the intra‐ and inter‐ICCs were 0.85 and 0.89, respectively. The T2 relaxation time significantly decreased from baseline (32.2 ± 2.9 ms) to 6 months (30.4 ± 3.4 ms, *p* < 0.001) and 12 months (29.4 ± 3.5 ms, *p* < 0.001) in the ESWT group while it remained unchanged in the sham treatment group (Table 3). There was a significant difference between the two groups in the mean change in T2 relaxation time from baseline to 12 months (ESWT, −2.9 ± 1.7 ms vs. control, 1.0 ± 1.9 ms; *p* < 0.001), indicating improvement of the meniscus degeneration in the ESWT group (Figure 3).

**Table 3:** Primary outcome data: T2 relaxation time.

	ESWT group (*n* = 14)	*p* Value^a^	Control group (*n* = 13)	*p* Value^a^
Pretreatment^b^	32.1 ± 2.9		30.2 ± 3.2	
6 months^b^	30.4 ± 3.4	<0.001	30.9 ± 3.3	n.s.
12 months^b^	29.4 ± 3.5	<0.001	31.2 ± 3.0	n.s.

Abbreviations: ESWT, extracorporeal shockwave therapy; n.s., not significant.

^a^ Paired‐sample *t*‐test with reference to pretreatment.

^b^ Values are presented as mean ± SD.

### Improvement of pain

**Figure 4:**
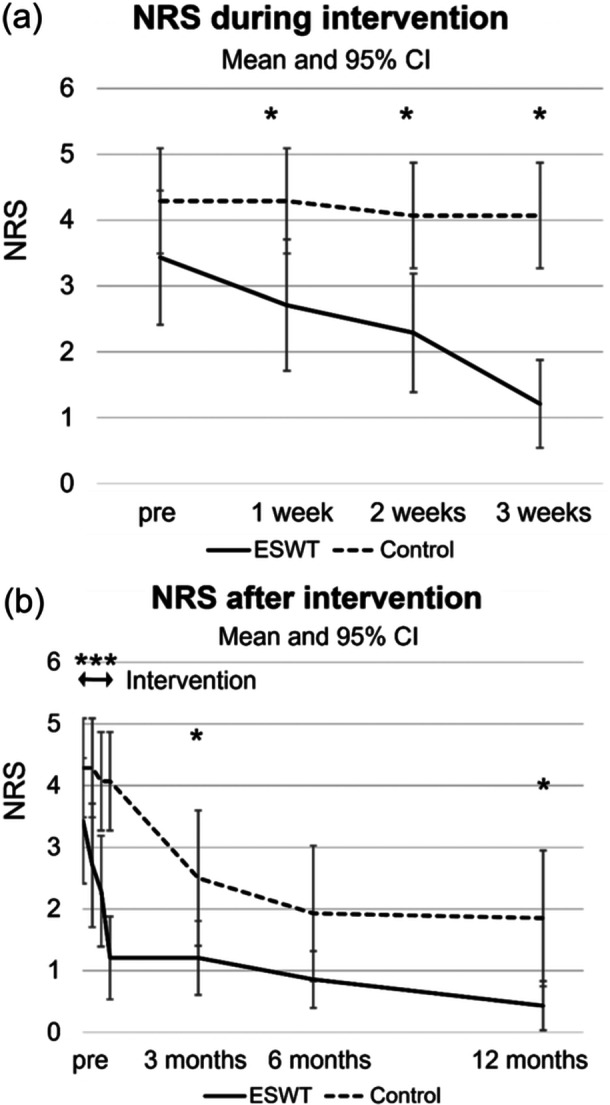
Short‐term and long‐term changes in pain. (a) numerical rating scale (NRS) of knee pain during the intervention: the extracorporeal shockwave therapy (ESWT) group showed significant pain improvement from the first treatment (1 week) to the third treatment (3 weeks). (b) NRS of knee pain during intervention; the ESWT group showed significant improvement in pain at 3 and 12 months post‐intervention.

The ESWT group showed significant improvement in knee pain during the 3‐week intervention sessions. The ESWT group also showed improvement in knee pain, which was significantly lower than that in the control group at 3 months (*p* = 0.042) and 12 months (*p* = 0.038) (Table 4 and Figure 4a,b). At the final follow‐up examination at 12 months, no patients in the ESWT group required analgesics, while three patients in the control group regularly used analgesics for knee pain (NSAIDs, *n* = 2; acetaminophen, *n* = 1).

**Table 4:** The numerical rating scale for knee pain and use of analgesics.

	ESWT group (*n* = 14)	Control group (*n* = 13)	*p* Value
NRS for knee pain			
Pretreatment	3.4 ± 1.9	4.3 ± 1.5	n.s.
1 week (first treatment)^a^	2.7 ± 1.9	4.3 ± 1.5	0.02
2 weeks (second treatment)^a^	2.3 ± 1.7	4.1 ± 1.6	0.01
3 weeks (third treatment)^a^	1.2 ± 1.3	4.1 ± 1.5	<0.001
3 months^a^	1.2 ± 1.1	2.5 ± 2.0	0.04
6 months^a^	0.9 ± 0.9	1.9 ± 1.9	n.s.
12 months^a^	0.4 ± 0.8	1.9 ± 2.1	0.04
Analgesics use			
Pretreatment	2 (14%)	2 (15%)	n.s.
3 months	1 (7%)	2 (15%)	n.s.
6 months	0 (0%)	3 (23%)	n.s.
12 months	0 (0%)	3 (23%)	n.s.

Abbreviations: NRS, numerical rating scale; n.s., not significant.

^a^ Values are presented as mean ± SD.

### Physical findings and PROs

At 12 months post‐intervention, the physical findings (ROM, patellar floating and McMurray test) of the two groups were similar (Table 5). All PROs in both groups improved significantly from baseline to 12 months post‐intervention (*p* < 0.001). The mean Lysholm score, IKDC score and KOOS were higher in the ESWT group than in the control group at 12 months after treatment, although the differences were not statistically significant (Table 5).

**Table 5:** Physical findings and patient‐reported outcomes at 12 months post‐treatment.

	ESWT group (*n* = 14)	Control group (*n* = 13)	*p* Value
ROM extension^a^ (°)	−0.7 ± 1.8	−2.3 ± 3.3	n.s.
ROM flexion^a^ (°)	137.5 ± 7.8	136.2 ± 3.6	n.s.
Patellar floating	1 (7%)	1 (8%)	n.s.
McMurray test	1 (7%)	5 (38%)	n.s.
IKDC^a^	75.3 ± 13.5	65.5 ± 14.8	n.s.
Lysholm score^a^	90.6 ± 10.6	86.4 ± 16.0	n.s.
KOOS			
Symptoms^a^	91.8 ± 11.9	86.1 ± 12.7	n.s.
Pain^a^	92.3 ± 10.4	85.5 ± 12.2	n.s.
ADL^a^	95.8 ± 6.2	93.7 ± 9.2	n.s.
Sports/Rec^a^	79.3 ± 25.3	73.5 ± 22.2	n.s.
QOL^a^	72.3 ± 22.0	68.8 ± 23.0	n.s.

Abbreviations: ADL, activities of daily living; ESWT, extracorporeal shockwave therapy; IKDC, International Knee Documentation Committee; KOOS, knee injury and osteoarthritis outcome score; n.s., not significant; QOL, quality of life; ROM, range of motion.

^a^ Values are presented as mean ± SD.

### Adverse events

No adverse events occurred during the study period in either of the groups.

## DISCUSSION

The most important finding of the present study was that ESWT led to a significant decrease in the T2 relaxation time in the degenerated meniscus at 12 months post‐intervention, thus indicating that it may improve the condition of the degenerated tissue. ESWT also provided pain relief at 12 months post‐intervention. The present study is the first randomized clinical trial to examine the efficacy of focused ESWT for the treatment of degenerative meniscal tears.

### Comparison with the relevant literature

Degenerative meniscal tear is a common finding in osteoarthritic knees; however, the current treatment is mostly limited to arthroscopic partial meniscectomy rather than regeneration of the injured meniscus [2, 19]. Recently, orthobiologics, such as platelet‐rich plasma and mesenchymal stem cells (MSCs), have emerged as potential therapeutics to improve meniscal healing [34]. Platelet‐rich plasma is clinically used for meniscal repair, although its efficacy remains controversial [14]. Satisfactory clinical results have been reported after the transplantation of synovial MSCs [36] and bone marrow‐derived MSCs [41] for degenerative tears. However, these treatments are difficult to implement in practice because of cost issues and limited clinical evidence. Therefore, several challenges remain in the treatment of degenerative meniscal tears. ESWT has the potential to be a simple, repeatable, safe and unique adjunctive treatment for the improvement of degenerative meniscus tears.

Different approaches for treating degenerative meniscus using external stimuli have also been intensively investigated, particularly in animal models. Low‐intensity pulsed ultrasound (LIPUS) has been reported to protect the meniscus from degenerative changes and promote tissue repair of meniscal tears in a rat model [20]. It was previously shown that ESWT, which provides a more potent stimulus to cells than LIPUS, has a therapeutic effect in a rat model of medial meniscal tears [13]. Furthermore, it was demonstrated that ESWT suppressed the progression of meniscal degeneration in a rat model of meniscal degeneration following anterior cruciate ligament tears [39]. In the present clinical study, ESWT was found to have a beneficial effect on degenerative meniscal tears. Regarding safety, no serious adverse events were observed in the patients treated with ESWT. Our results demonstrate that ESWT is a potential adjunct treatment for degenerative meniscal tears.

ESWT is known to have regenerative effects on various tissues, including tendons, ligaments and bones [6, 10, 12, 17, 22, 37, 40]. The efficacy of ESWT is due to the direct stimulation of the cells and can be ascribed to the transduction of the acoustic shockwave signal into biological signals that result in cell proliferation or differentiation through a mechano‐transduction process [10, 37]. ESWT promotes tissue regeneration via the upregulation of gene expression, such as vessel endothelial growth factor, proliferating cell nuclear antigen, transforming growth factor‐beta1 and bone morphogenetic proteins [18, 42, 43]. In our previous research using a rat model, we showed that a reparative effect of ESWT was facilitated by cartilage‐specific genes [13, 39]. We found that ESWT exerts its therapeutic effect through the upregulation of the cartilage repair factors SRY‐box transcription factor 9 (SOX9) and cellular communication network factor 2 (CCN2). Additionally, ESWT promoted the cellular activity and mRNA expression of collagen type 2. In accordance with our findings, a previous study in a rabbit model reported that ESWT increased the expression of SOX9 during the healing of tendon bone insertion [7].

The present study was conducted using the ESWT intervention protocol recommended by the International Society for Medical Shockwave Treatment (0.25 mJ/mm^2^, 2000 impulses and 3 sessions of treatment with a 1‐week interval). However, the optimal protocol for ESWT remains unclear. ESWT has been reported to have different therapeutic effects depending on the intensity of energy and number of impulses and sessions in various diseases [11, 48]. Whereas protocol modifications, such as the number of sessions or impulses, promote a reduction in T2 relaxation time and improved clinical outcomes remain open to investigation.

In this study, patients who received ESWT experienced immediate and long‐lasting pain relief. The pain‐relieving effect of ESWT is considered to be mainly due to the denervation of the free endings of pain‐inducing sensory nerves [15, 33]. ESWT has also been shown to inhibit two pain transmitters (calcitonin gene‐related peptide and substance‐P) in a rat model [16, 21, 32, 38]. In contrast to the relatively established role of ESWT in short‐term pain relief, its long‐term effects remain much less understood. Previous clinical studies have produced mixed results in terms of long‐term pain after ESWT [4, 8]. Further research on the underlying mechanism is needed.

The present study is associated with several limitations. First, in this exploratory study, the biological effects of ESWT were evaluated using quantitative MRI. Although a difference in the primary outcome (i.e., change in the T2 relaxation time) was successfully detected, the study had insufficient statistical power to validate the clinical effectiveness of ESWT. A larger study with a primary clinical endpoint is required to confirm the effectiveness of ESWT. Second, complete masking was not possible because ESWT caused minimal pain during the delivery. Finally, we were unable to standardize the rehabilitation programme for patients. However, we assumed that the variations were minimal because the trial was conducted by a team at a single institution. Third, In the context of T2 mapping for cartilage and meniscus assessment, many studies have utilized 3 T MRI. However, at our facility, such equipment was unavailable. While there are studies evaluating meniscal treatment or cartilage using 1.5 T MRI [3, 23, 27], the potential impact of equipment differences on our current results cannot be disregarded.

## CONCLUSION

In this exploratory randomized trial involving symptomatic patients with degenerative meniscal tears, ESWT led to a significant decrease in the T2 relaxation time on T2 mapping in the degenerative meniscus, indicating that it may improve the condition of the meniscus. Our results demonstrate that ESWT may, therefore, be a potentially effective adjunct treatment for degenerative meniscal tears.

## AUTHOR CONTRIBUTIONS

**Shogo Hashimoto**: Conceptualization; data curation; formal analysis; funding acquisition; investigation; methodology; visualization; writing—original draft. **Takashi Ohsawa**: Conceptualization; methodology. **Hiroaki Omae**: Investigation. **Atsufumi Oshima**: Validation. **Ryota Takase**: Investigation. **Hirotaka Chikuda**: Supervision; writing—review and editing.

## CONFLICT OF INTEREST STATEMENT

This study borrowed the extracorporeal shockwave equipment from KARL STORZ Endoscopy Japan K.K. The company had no role in the design, conduct of the study, analysis, or interpretation of the data.

## ETHICS STATEMENT

The study protocol was approved by the institutional review board of Gunma University (jRCTs032200082). All participants were informed of the purpose and content of the study and provided their written informed consent.

## Supporting information

Supplementary information.

Supplementary information.

## Data Availability

The datasets generated and analysed during the current study are not publicly available because access to the information management system we are using is restricted to authorized personnel only. However, they are available from the corresponding author upon reasonable request.

## References

[1] Baum, T. , Joseph, G.B. , Karampinos, D.C. , Jungmann, P.M. , Link, T.M. & Bauer, J.S. (2013) Cartilage and meniscal T2 relaxation time as non‐invasive biomarker for knee osteoarthritis and cartilage repair procedures. Osteoarthritis and Cartilage, 21, 1474–1484. Available from: 10.1016/j.joca.2013.07.012 23896316 PMC3929642

[2] Beaufils, P. , Becker, R. , Kopf, S. , Englund, M. , Verdonk, R. , Ollivier, M. et al. (2017) Surgical management of degenerative meniscus lesions: the 2016 ESSKA meniscus consensus. Knee Surgery, Sports Traumatology, Arthroscopy, 25, 335–346. Available from: 10.1007/s00167-016-4407-4 PMC5331096 28210788

[3] Berton, A. , Longo, U.G. , Candela, V. , Greco, F. , Martina, F.M. , Quattrocchi, C.C. et al. (2020) Quantitative evaluation of meniscal healing process of degenerative meniscus lesions treated with hyaluronic acid: a clinical and MRI study. Journal of Clinical Medicine, 9, 2280. Available from: 10.3390/jcm9072280 32709084 PMC7408658

[4] Carulli, C. , Tonelli, F. , Innocenti, M. , Gambardella, B. , Muncibì, F. & Innocenti, M. (2016) Effectiveness of extracorporeal shockwave therapy in three major tendon diseases. Journal of Orthopaedics and Traumatology, 17, 15–20. Available from: 10.1007/s10195-015-0361-z 26135551 PMC4805637

[5] Challen, J. , Tang, Y. , Hazratwala, K. & Stuckey, S. (2007) Accuracy of MRI diagnosis of internal derangement of the knee in a non‐specialized tertiary level referral teaching hospital. Australasian Radiology, 51, 426–431. Available from: 10.1111/j.1440-1673.2007.01865.x 17803793

[6] Cheng, J.H. & Wang, C.J. (2015) Biological mechanism of shockwave in bone. International Journal of Surgery, 24, 143–146. Available from: 10.1016/j.ijsu.2015.06.059 26118613

[7] Chow, D.H.K. , Suen, P.K. , Huang, L. , Cheung, W.H. , Leung, K.S. , Ng, C. et al. (2014) Extracorporeal shockwave enhanced regeneration of fibrocartilage in a delayed tendon‐bone insertion repair model. Journal of Orthopaedic Research, 32, 507–514. Available from: 10.1002/jor.22566 24375544

[8] Chung, B. , Wiley, J.P. & Rose, M.S. (2005) Long‐term effectiveness of extracorporeal shockwave therapy in the treatment of previously untreated lateral epicondylitis. Clinical Journal of Sport Medicine, 15, 305–312. Available from: 10.1097/01.jsm.0000179137.69598.7e 16162988

[9] Eijgenraam, S.M. , Bovendeert, F.A.T. , Verschueren, J. , van Tiel, J. , Bastiaansen‐Jenniskens, Y.M. , Wesdorp, M.A. et al. (2019) T(2) mapping of the meniscus is a biomarker for early osteoarthritis. European Radiology, 29, 5664–5672. Available from: 10.1007/s00330-019-06091-1 30888480 PMC6719322

[10] Frairia, R. & Berta, L. (2011) Biological effects of extracorporeal shock waves on fibroblasts. Muscles, Ligaments and Tendons Journal, 1, 138–147. 23738262 PMC3666484

[11] Gezginaslan, Ö. & Başar, G. (2021) Comparison of Effectiveness of density and number of sessions of extracorporeal shock wave therapy in plantar fasciitis patients: a double‐blind, randomized‐controlled study. The Journal of Foot and Ankle Surgery, 60, 262–268. Available from: 10.1053/j.jfas.2020.08.001 33191061

[12] Gollwitzer, H. , Saxena, A. , DiDomenico, L.A. , Galli, L. , Bouché, R.T. , Caminear, D.S. et al. (2015) Clinically relevant effectiveness of focused extracorporeal shock wave therapy in the treatment of chronic plantar fasciitis: a randomized, controlled multicenter study. The Journal of Bone and Joint Surgery‐American Volume, 97, 701–708. Available from: 10.2106/JBJS.M.01331 25948515

[13] Hashimoto, S. , Ichinose, T. , Ohsawa, T. , Koibuchi, N. & Chikuda, H. (2019) Extracorporeal shockwave therapy accelerates the healing of a meniscal tear in the avascular region in a rat model. The American Journal of Sports Medicine, 47, 2937–2944. Available from: 10.1177/0363546519871059 31503505

[14] Haunschild, E.D. , Huddleston, H.P. , Chahla, J. , Gilat, R. , Cole, B.J. & Yanke, A.B. (2020) Platelet‐rich plasma augmentation in meniscal repair surgery: a systematic review of comparative studies. Arthroscopy: The Journal of Arthroscopic & Related Surgery, 36, 1765–1774. Available from: 10.1016/j.arthro.2020.01.038 32057981

[15] Hausdorf, J. , Lemmens, M.A.M. , Heck, K.D.W. , Grolms, N. , Korr, H. , Kertschanska, S. et al. (2008) Selective loss of unmyelinated nerve fibers after extracorporeal shockwave application to the musculoskeletal system. Neuroscience, 155, 138–144. Available from: 10.1016/j.neuroscience.2008.03.062 18579315

[16] Hausdorf, J. , Lemmens, M.A.M. , Kaplan, S. , Marangoz, C. , Milz, S. , Odaci, E. et al. (2008) Extracorporeal shockwave application to the distal femur of rabbits diminishes the number of neurons immunoreactive for substance P in dorsal root ganglia L5. Brain Research, 1207, 96–101. Available from: 10.1016/j.brainres.2008.02.013 18371941

[17] Ho, K.D. , Yang, C.L. , Lo, H.Y. & Yeh, H.J. (2022) Extracorporeal shockwave therapy with a modified technique on tendon and ligament for knee osteoarthritis: a randomized controlled trial. American Journal of Physical Medicine & Rehabilitation, 101, 11–17. Available from: 10.1097/PHM.0000000000001730 34915541

[18] Hofmann, A. , Ritz, U. , Hessmann, M.H. , Alini, M. , Rommens, P.M. & Rompe, J.D. (2008) Extracorporeal shock wave‐mediated changes in proliferation, differentiation, and gene expression of human osteoblasts. Journal of Trauma: Injury, Infection & Critical Care, 65, 1402–1410. Available from: 10.1097/TA.0b013e318173e7c2 19077634

[19] Hohmann, E. (2023) Treatment of degenerative meniscus tears. Arthroscopy: The Journal of Arthroscopic & Related Surgery, 39, 911–912. Available from: 10.1016/j.arthro.2022.12.002 36872031

[20] Kamatsuki, Y. , Aoyama, E. , Furumatsu, T. , Miyazawa, S. , Maehara, A. , Yamanaka, N. et al. (2018) Possible reparative effect of low‐intensity pulsed ultrasound (LIPUS) on injured meniscus. Journal of Cell Communication and Signaling, 13, 193–207. Available from: 10.1007/s12079-018-0496-9 30460593 PMC6498275

[21] Lian, Ø. , Dahl, J. , Ackermann, P.W. , Frihagen, F. , Engebretsen, L. & Bahr, R. (2006) Pronociceptive and antinociceptive neuromediators in patellar tendinopathy. The American Journal of Sports Medicine, 34, 1801–1808. Available from: 10.1177/0363546506289169 16816149

[22] Liao, P.C. , Chou, S.H. & Shih, C.L. (2024) A systematic review of the use of shockwave therapy for knee osteoarthritis. Journal of Orthopaedics, 56, 18–25. Available from: 10.1016/j.jor.2024.04.020 38765896 PMC11096685

[23] Liess, C. , Lüsse, S. , Karger, N. , Heller, M. & Glüer, C.C. (2002) Detection of changes in cartilage water content using MRI T2‐mapping in vivo. Osteoarthritis and Cartilage, 10, 907–913. Available from: 10.1053/joca.2002.0847 12464550

[24] Liu, F. , Samsonov, A. , Wilson, J.J. , Blankenbaker, D.G. , Block, W.F. & Kijowski, R. (2015) Rapid in vivo multicomponent T2 mapping of human knee menisci. Journal of Magnetic Resonance Imaging, 42, 1321–1328. Available from: 10.1002/jmri.24901 25847733 PMC4880357

[25] Luvsannyam, E. , Jain, M.S. , Leitao, A.R. , Maikawa, N. & Leitao, A.E. (2022) Meniscus tear: pathology, incidence, and management. Cureus, 14, e25121. Available from: 10.7759/cureus.25121. 35733484 PMC9205760

[26] López‐Franco, M. & Gómez‐Barrena, E. (2018) Cellular and molecular meniscal changes in the degenerative knee: a review. Journal of Experimental Orthopaedics, 5, 11. Available from: 10.1186/s40634-018-0126-8 29675769 PMC5908770

[27] Mars, M. , Chelli, M. , Tbini, Z. , Ladeb, F. & Gharbi, S. (2018) MRI T2 mapping of knee articular cartilage using different acquisition sequences and calculation methods at 1.5 Tesla. Medical Principles and Practice, 27, 443–450. Available from: 10.1159/000490796 29895028 PMC6243913

[28] McCauley, T.R. (2005) MR imaging evaluation of the postoperative knee. Radiology, 234, 53–61. Available from: 10.1148/radiol.2341031302 15564389

[29] Nebelung, S. , Tingart, M. , Pufe, T. , Kuhl, C. , Jahr, H. & Truhn, D. (2016) Ex vivo quantitative multiparametric MRI mapping of human meniscus degeneration. Skeletal Radiology, 45, 1649–1660. Available from: 10.1007/s00256-016-2480-x 27639388

[30] Nishino, K. , Hashimoto, Y. , Nishida, Y. , Yamasaki, S. & Nakamura, H. (2023) Arthroscopic surgery for symptomatic discoid lateral meniscus improves meniscal status assessed by magnetic resonance imaging T2 mapping. Archives of Orthopaedic and Trauma Surgery, 143, 4889–4897. Available from: 10.1007/s00402-023-04819-9 36811665

[31] Nishioka, H. , Hirose, J. , Nakamura, E. , Oniki, Y. , Takada, K. , Yamashita, Y. et al. (2012) T1ρ and T2 mapping reveal the in vivo extracellular matrix of articular cartilage. Journal of Magnetic Resonance Imaging, 35, 147–155. Available from: 10.1002/jmri.22811 21990043

[32] Ochiai, N. , Ohtori, S. , Sasho, T. , Nakagawa, K. , Takahashi, K. , Takahashi, N. et al. (2007) Extracorporeal shock wave therapy improves motor dysfunction and pain originating from knee osteoarthritis in rats. Osteoarthritis and Cartilage, 15, 1093–1096. Available from: 10.1016/j.joca.2007.03.011 17466542

[33] Ohtori, S. , Inoue, G. , Mannoji, C. , Saisu, T. , Takahashi, K. , Mitsuhashi, S. et al. (2001) Shock wave application to rat skin induces degeneration and reinnervation of sensory nerve fibres. Neuroscience Letters, 315, 57–60. Available from: 10.1016/S0304-3940(01)02320-5 11711214

[34] Ozeki, N. , Koga, H. & Sekiya, I. (2022) Degenerative meniscus in knee osteoarthritis: from pathology to treatment. Life, 12, 603. Available from: 10.3390/life12040603 35455094 PMC9032096

[35] Russo, A. , Capasso, R. , Varelli, C. , Laporta, A. , Carbone, M. , D'Agosto, G. et al. (2017) MR imaging evaluation of the postoperative meniscus. Musculoskeletal Surgery, 101, 37–42. Available from: 10.1007/s12306-017-0454-3 28210945

[36] Sekiya, I. , Koga, H. , Otabe, K. , Nakagawa, Y. , Katano, H. , Ozeki, N. et al. (2019) Additional use of synovial mesenchymal stem cell transplantation following surgical repair of a complex degenerative tear of the medial meniscus of the knee: a case report. Cell Transplantation, 28, 1445–1454. Available from: 10.1177/0963689719863793 31313604 PMC6802148

[37] Simplicio, C.L. , Purita, J. , Murrell, W. , Santos, G.S. , Dos Santos, R.G. & Lana, J.F.S.D. (2020) Extracorporeal shock wave therapy mechanisms in musculoskeletal regenerative medicine. Journal of Clinical Orthopaedics and Trauma, 11, S309–S318. Available from: 10.1016/j.jcot.2020.02.004 32523286 PMC7275282

[38] Takahashi, N. , Wada, Y. , Ohtori, S. , Saisu, T. & Moriya, H. (2003) Application of shock waves to rat skin decreases calcitonin gene‐related peptide immunoreactivity in dorsal root ganglion neurons. Autonomic Neuroscience, 107, 81–84. Available from: 10.1016/S1566-0702(03)00134-6 12963418

[39] Takase, R. , Ichinose, T. , Hashimoto, S. , Amano, I. , Ohsawa, T. , Koibuchi, N. et al. (2024) Protective effects of extracorporeal shockwave therapy on the degenerated meniscus in a rat model. The American Journal of Sports Medicine, 52, 374–382. Available from: 10.1177/03635465231214697 38174366

[40] Tang, P. , Wen, T. , Lu, W. , Jin, H. , Pan, L. , Li, H. et al. (2024) The efficacy of extracorporeal shock wave therapy for knee osteoarthritis: an umbrella review. International Journal of Surgery, 110, 2389–2395. Available from: 10.1097/JS9.0000000000001116 38668665 PMC11020044

[41] Vangsness, Jr., C.T. , Farr, 2nd, J. , Boyd, J. , Dellaero, D.T. , Mills, C.R. & LeRoux‐Williams, M. (2014) Adult human mesenchymal stem cells delivered via intra‐articular injection to the knee following partial medial meniscectomy: a randomized, double‐blind, controlled study. Journal of Bone and Joint Surgery, 96, 90–98. Available from: 10.2106/JBJS.M.00058 24430407

[42] Wang, C.J. (2012) Extracorporeal shockwave therapy in musculoskeletal disorders. Journal of Orthopaedic Surgery and Research, 7, 11. Available from: 10.1186/1749-799X-7-11 22433113 PMC3342893

[43] Wang, F.S. , Yang, K.D. , Chen, R.F. , Wang, C.J. & Sheen‐Chen, S.M. (2002) Extracorporeal shock wave promotes growth and differentiation of bone‐marrow stromal cells towards osteoprogenitors associated with induction of TGF‐beta1. The Journal of Bone and Joint Surgery. British Volume, 84, 457–461. Available from: 10.1302/0301-620x.84b3.11609 12002511

[44] Warnecke, D. , Balko, J. , Haas, J. , Bieger, R. , Leucht, F. , Wolf, N. et al. (2020) Degeneration alters the biomechanical properties and structural composition of lateral human menisci. Osteoarthritis and Cartilage, 28, 1482–1491. Available from: 10.1016/j.joca.2020.07.004 32739340

[45] White, L.M. , Schweitzer, M.E. , Weishaupt, D. , Kramer, J. , Davis, A. & Marks, P.H. (2002) Diagnosis of recurrent meniscal tears: prospective evaluation of conventional MR imaging, indirect MR arthrography, and direct MR arthrography. Radiology, 222, 421–429. Available from: 10.1148/radiol.2222010396 11818609

[46] Wuerfel, T. , Schmitz, C. & Jokinen, L.L.J. (2022) The effects of the exposure of musculoskeletal tissue to extracorporeal shock waves. Biomedicines, 10, 1084. Available from: 10.3390/biomedicines10051084 35625821 PMC9138291

[47] Yamasaki, S. , Hashimoto, Y. , Nishida, Y. , Teraoka, T. , Terai, S. , Takigami, J. et al. (2020) Assessment of meniscal healing status by magnetic resonance imaging t2 mapping after meniscal repair. The American Journal of Sports Medicine, 48, 853–860. Available from: 10.1177/0363546520904680 32167835

[48] Zhang, Y.F. , Liu, Y. , Chou, S.W. & Weng, H. (2021) Dose‐related effects of radial extracorporeal shock wave therapy for knee osteoarthritis: a randomized controlled trial. Journal of Rehabilitation Medicine, 53, jrm00144. Available from: 10.2340/16501977-2782 33367924 PMC8772366

