# Supplementary material for: Extracorporeal shockwave therapy for degenerative meniscal tears results in a decreased T2 relaxation time and pain relief: An exploratory randomized clinical trial
Source: Knee Surg Sports Traumatol Arthrosc. 2024 Aug 5;32(12):3141–50. doi: 10.1002/ksa.12384 (PMC11605018; doi:10.1002/ksa.12384)
Supplement: Supplementary file 2 — Supplementary information. [file KSA-32-3141-s001.docx]

# **Implementation Plan**

**Since we would like to conduct specific clinical research as follows, we submit an implementation plan in accordance with the provisions of Article 5, Paragraph 1 of the Clinical Trials Act.**

## **1 Matters related to the implementation system for specified clinical research and matters related to the structural equipment of facilities for conducting specified clinical research**

**(1) Name of research**

| **Scientific Title（Acronym）** | **Clinical study on therapeutic effect of extracorporeal shockwave therapy for meniscal degenerative tear** |
| --- | --- |
| **Public Title（Acronym）** | **Therapeutic effect of extracorporeal shockwave therapy for meniscal degenerative tear** |

**(2) Matters related to the principal investigator (in the case of multicenter collaborative research, the principal investigator)**

**Omitted for blinding.**

**(3) Matters related to persons engaged in clinical research other than the principal investigator**

**Omitted for blinding.**

**(4) Matters related to the principal investigator in multicenter joint research**

**Omitted for blinding.**

## **2. Outline of the purpose and content of the specified clinical research and the pharmaceuticals used therein**

**(1) Purpose and content of specific clinical research**

| **Research Objectives** | | **Investigating the therapeutic effects of extracorporeal shockwave therapy on meniscus** **degenerative tear** | |
| --- | --- | --- | --- |
| **Phase** | | **PhaseⅠ～Ⅱ** | |
| **Scheduled start date of patient registration** | | **jRCT Release Date** | |
| **Implementation Period** | | **jRCT release date~2022**/**9/30** | |
| **Number of** **research subjects** **to be conducted** | | **30 people** | |
| **Study Type** | | **Intervention** | |
| **Study Design** | | **Randomized controlled trial**  **Single-blind**  **Placebo control**  **Parallel assignment**  **Treatment purpose** | |
| **With or without placebo** | | **■ Yes** | **□ None** |
| **Blinding** | | **■ Yes** | **□ None** |
| **Presence or absence of randomization** | | **■ Yes** | **□ None** |
| **Whether or not there is combined medical treatment outside of insurance** | | **■ Yes** | **□ None** |
| **Countries where clinical research is conducted (non-Japan)** | | **without** | |
| **Countries of Recruitment** | | **none** | |
|  | **Inclusion Criteria** | **1) Patients over 40 years old**  **2) Patients diagnosed with meniscal degeneration by MRI**  **3) Patients with knee pain above NRS2**  **4) Patients with PS between 0 and 1**  **5) Outpatient**  **6) Patients obtained from consent documents** | |
|  | **Exclusion Criteria** | **1) Patients with knee osteoarthritis of G3 or higher according to the Kellgren-Lawrence classification**  **2) Patients diagnosed with cognitive decline at the physician's discretion**  **3) Patients with untreated blood coagulation disorders such as hemophilia or patients taking anticoagulants**  **4) Patients with thrombosis**  **5) Patients with malignant tumor**  **6) Pregnant woman**  **7) Patients who have received steroid treatment for 6 months or more, or within 6 weeks after steroid injection**  **8) Patients judged inappropriate by the doctor** | |
|  | **Age Minimum** | **40** | |
|  | **Age Maximum** | **not applicable** | |
|  | **Gender** | **Both** | |
| **Discontinuation criteria** | | **Surgical Procedures** | |
| **Health Condition(s) or Problem(s) Studied** | | **Meniscal degenerative tear** | |
| **Presence or absence of intervention** | | **■ Yes** | **□ None** |
| **Details of the intervention** | | **Extracorporeal shockwave therapy** | |
| **Intervention(s)** | | **Extracorporeal shockwave therapy** | |
| **Primary Outcome(s)** | | **T2 value in MRI** | |
| **Secondary Outcome(s)** | | **NRS**  **Clinical score（KOOS, IKDC score, Lysholme score）**  **Scientific findings（Ballottement of patella, Range of motion, Mcmurray test, Watson-Johns test）**  **Adverse event**  **Daily use of oral analgesics**  **Transition rate to surgery after starting treatment** | |

**(2) Outline of pharmaceuticals, etc. used in specified clinical research**

| **Pharmaceuticals, Medical Devices, Regenerative Medicine Products** | | | **□ Pharmaceuticals** | **■ Medical Devices** | **□ Regenerative medicine products** |
| --- | --- | --- | --- | --- | --- |
| **Not approved under the Pharmaceuticals and Medical Devices Act,**  **Off-label, different within approval** | | | **□ Unapproved** | **■ Off Label** | **□ Within approval** |
| **General name, etc.** | **medicine** | **General name (if not approved in Japan or overseas, the development code must be indicated)** | **n/a** | | |
|  |  | **Product name (for overseas products, the country name must also be indicated)** | **n/a** | | |
|  |  | **Grant Number** | **n/a** | | |
|  | **Medical Devices** | **classification** | **Machinery and Apparatus 12 Physical Practice Instruments** | | |
|  |  | **Generic name** | **extracorporeal shock wave pain therapy device** | | |
|  |  | **Approval, Certification, and Notification Number** | **71029000** | | |
|  | **Regenerative Medicine Products** | **classification** | **n/a** | | |
|  |  | **Generic name** | **n/a** | | |
|  |  | **Grant Number** | **n/a** | | |
| **Drug Providers** | | **name** | **Omitted for blinding.** | | |
|  |  | **location** | **Omitted for blinding.** | | |

## **3 Matters related to confirmation of the implementation status of specific clinical research**

**(1) Schedule of audits**

| **Whether audits are planned to be conducted** | **□ Yes** | **■ None** |
| --- | --- | --- |

## **4 Matters related to compensation and provision of medical care in the event of health damage to the subject of the specified clinical research**

| **Whether or not there is compensation for subjects of specific clinical studies** | | | **□ Yes** | **■ None** |
| --- | --- | --- | --- | --- |
| **compensation**  **Contents** | **Insurance coverage** | | **□ Yes** | **■ None** |
|  |  | **Insurance coverage** |  | |
|  | **Non-insurance coverage** | | **none** | |

## **5 Matters concerning the involvement of pharmaceutical manufacturers, distributors who manufacture and sell pharmaceuticals, etc. used in specified clinical research and their special related parties in the specified clinical research**

**(1) Provision of research funds, etc. from manufacturers and distributors of pharmaceuticals, etc. such as pharmaceuticals used for specified clinical research**

| **Name of pharmaceutical manufacturer, etc. that manufactures and sells pharmaceuticals, etc. used for specified clinical research, or intends to do so** | | **Omitted for blinding.** | |
| --- | --- | --- | --- |
| **Whether or not research funds are provided** | | **□ Yes** | **■ None** |
| **Name of Research Funding Organization** | |  | |
| **Source of Monetary Support/**  **Secondary Sponsor** | |  | |
| **Secondary Sponsor Applicability** | | **□ Yes** | **□ Not applicable** |
| **Whether or not a contract for the provision of research funds, etc. has been concluded** | | **□ Yes** | **□ None** |
|  | **Date of contract conclusion** |  | |
| **Provision of goods** | | **■ Yes** | **□ None** |
|  | **Details of provision of goods** | **extracorporeal shock wave therapy device** | |
| **Provision of services** | | **□ Yes** | **■ None** |
|  | **Details of service provision** |  | |

*** If more than one applies, copy the above items and write them down.**

**(2) Provision of research funds from other than manufacturers and distributors of pharmaceuticals, such as pharmaceuticals used for specified clinical research,**

| **Whether or not research funds are provided** | **□ Yes** | **■ None** |
| --- | --- | --- |
| **Name of Research Funding Organization** |  | |
| **Source of Monetary Support** |  | |
| **Secondary Sponsor Applicability** | **□ Yes** | **■ Not applicable** |

*** If more than one applies, copy the above items and write them down.**

## **6 Name of the Accredited Clinical Research Review Committee, etc. that conducts review and opinion services**

**Omitted for blinding.**
